# Demyelinating neuropathy as the initial presentation of familial E200K Creutzfeldt–Jakob disease in two patients

**DOI:** 10.1002/acn3.52296

**Published:** 2025-01-12

**Authors:** Cécile Delorme, Antoine Pégat, Julian Theuriet, Jean‐Philippe Brandel, Emmanuel Roze, Karine Viala, Julie Zyss, Stéphane Thobois, Anthony Fourier, Emilien Bernard, Juliette Svahn, Chloé Laurencin, Paul Jaulent, Christophe Vandendries, Isabelle Quadrio, Virginie Desestret, David Meyronet, Thierry Maisonobe, Stéphane Haïk, Danielle Seilhean

**Affiliations:** ^1^ Service de Neurologie Hôpital Pitié‐Salpêtrière AP‐HP Paris France; ^2^ Service ENMG et de Pathologies Neuromusculaires, Centre de Référence des Maladies Neuromusculaires PACA‐Réunion‐Rhône Alpes Hôpital Neurologique Pierre Wertheimer, Hospices Civils de Lyon Bron France; ^3^ Service de Neurologie C, Pathologies du Mouvement et Pathologies Neuromusculaires Hôpital Neurologique Pierre Wertheimer, Hospices Civils de Lyon Bron France; ^4^ Cellule Nationale de Référence des Maladies de Creutzfeldt‐Jakob AP‐HP‐Sorbonne Université Paris France; ^5^ Institut du Cerveau et de la Moelle Épinière, ICM Sorbonne Université, INSERM, CNRS Paris France; ^6^ Département de Neurophysiologie Clinique Hôpital Pitié‐Salpêtrière AP‐HP Paris France; ^7^ Unité de Diagnostic des Pathologies Dégénératives, Biochimie et Biologie Moléculaire – LBBMS Centre de Biologie et Pathologie Est Groupement Est Bron France; ^8^ Centre Médical RMX Paris France; ^9^ Service de Neurocognition et Neuro‐ophtalmologie Hôpital Neurologique Pierre Wertheimer, Hospices Civils de Lyon Bron France; ^10^ Service D'Anatomie et de Cytologie Pathologique Centre de biologie et pathologie Est Groupement Est Bron France; ^11^ Département de Neuropathologie Raymond Escourolle Hôpital Pitié‐Salpêtrière, AP‐HP‐Sorbonne Université Paris France

## Abstract

**Objective:**

To describe peripheral neuropathy associated with familial Creutzfeldt‐Jakob disease.

**Methods:**

We report two unrelated patients with genetic Creutzfeldt–Jakob disease with demyelinating peripheral neuropathy as initial presentation, with a comprehensive clinical, electrophysiological and neuropathological description.

**Results:**

Both patients exhibited gait disturbance and paresthesia. Electrodiagnostic studies revealed demyelinating abnormalities with motor conduction blocks suggestive of chronic inflammatory demyelinating polyradiculoneuropathy, with abnormal plexus MRI and elevated CSF protein levels. One of them had *pes cavus* and a late‐onset Charcot–Marie‐Tooth (CMT) disease was also initially hypothesized. Central nervous system involvement manifested 1–2 years after the onset of peripheral symptoms. Both patients had a heterozygous E200K mutation in the *PRNP* gene. Postmortem neuropathological examinations showed PrP^Sc^ deposits in the peripheral nervous system, particularly in Schwann cells. **Interpretation**: Peripheral neuropathy in E200K genetic forms of Creutzfeldt‐Jakob disease can be inaugural and mimic chronic inflammatory demyelinating polyradiculoneuropathy.

## Introduction

Creutzfeldt–Jakob disease (CJD) is a fatal human spongiform encephalopathy caused by the aggregation of the misfolded isoform (PrP^Sc^) of the host‐encoded prion protein (PrP^C^). Familial forms are caused by heterozygous mutations in the *PRNP* gene, the E200K pathogenic variant (c.598G>A [p.Glu200Lys]) being the most frequent in Europe.^1^

While CJD primarily affects the central nervous system (CNS), it can also involve the peripheral nervous system (PNS).^2^, ^3^ Genetic forms of CJD due to the E200K mutation may present with peripheral neuropathy.^4^, ^5^, ^6^, ^7^ Demyelinating neuropathies mimicking chronic inflammatory demyelinating polyradiculoneuropathy (CIDP) have been described, both in familial and sporadic forms.^6^, ^8^, ^9^ Features of peripheral neuropathy were concomitant with the CNS manifestations, or preceded CNS symptoms by a few months in some cases.^6^, ^8^ None of the previous studies investigated in detail the clinical, electrophysiological, neuroimaging, and neuropathological features of PNS involvement in CJD.

We report the detailed characteristics of two new unrelated patients with CJD carrying the E200K mutation, for whom the initial presentation was a demyelinating neuropathy mimicking CIDP.

## Methods

The patients were admitted in two neuromuscular diseases centers in France. Electrodiagnostic study comprised nerve conduction studies and electromyography using a concentric needle electrode. All procedures adhered to the ethical standards (ethical approval 23_258) in accordance with the 1964 Helsinki declaration. Informed consents were obtained from family members of the deceased patients. Central and peripheral nerve tissue samples were obtained postmortem, after consent from next of kin and inclusion in the national Creutzfeldt–Jakob disease surveillance network, supported by *Santé Publique France*. Neuropathological examination followed the protocol already described, adding samples of dorsal root ganglia (DRG; patients 1 and 2), spinal cord, and peripheral nerves (patient 1). PrP immunohistochemistry (Bertin Pharma, Montigny le Bretonneux, France; 12F10, 1/200) was performed in all regions examined.^10^ In case 1, double labeling of the samples from spinal cord, DRG, and peripheral nerves was performed by combining the anti‐PrP antibody with an anti‐neurofilament (Dako, Santa Clara, CA, USA; 2F11, 1/2000) for nerve fibers, or an anti‐S100 protein antibody (Dako, rabbit polyclonal, prediluted) for Schwann cells, using diaminobenzidine (DAB) and alkaline phosphatase (ALP) as chromogens, brown and red, respectively.

## Results

**Figure 1:**
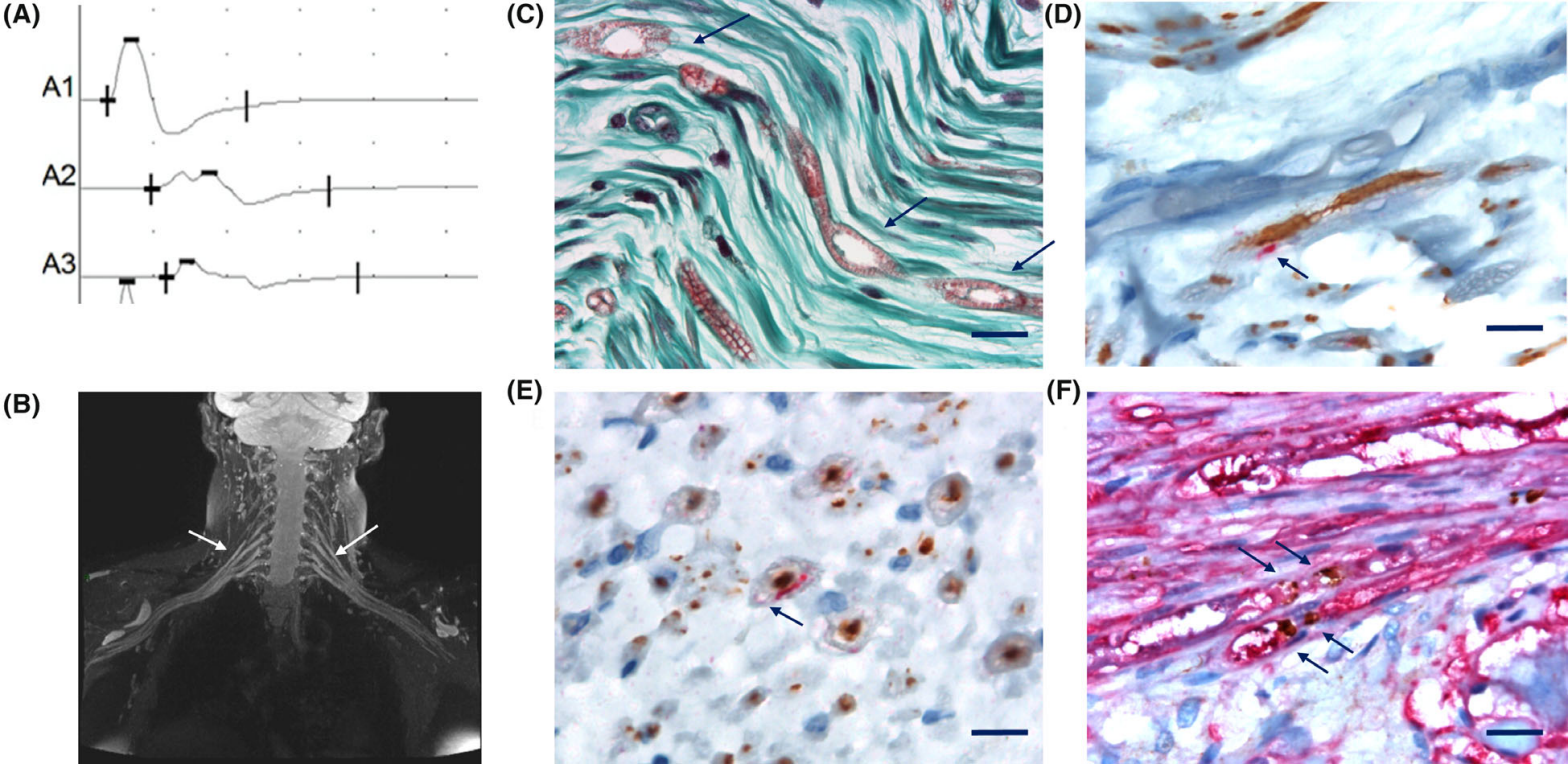
Paraclinical findings of patient 1. (A) Nerve conduction study (patient 1), 5 mV‐10 ms/div: motor nerve conduction study of the median nerve: (A1) at the wrist, (A2) at the elbow, (A3) at middle level of the arm. There was a conduction block (70% reduction of amplitude) between the wrist and the elbow. (B) Brachial plexus MRI (patient 1), coronal plane, conventional T2‐weighted sequences with STIR suppression. Bilateral diffuse hyperintensity and mild hypertrophy of the brachial plexus. (C–E) Superficial fibular nerve postmortem sample (patient 1). (C) Masson's trichrome stain showing myelin sheath alterations (arrows); (D and E) Double‐labelling of neurofilament (brown) and PrP^Sc^ (red) showing localization of PrP^Sc^ in the myelin sheath. (D) Longitudinal section; (E) transversal section. Scale bars = 10 μm. (F) Dorsal root ganglia postmortem sample (patient 1): Double labeling of Schwann cells (S100 protein in red) with detection of PrP^Sc^ (brown) on either side of the nodes of Ranvier (arrows). Scale bar = 10 μm.

Patient 1 was in her late fifties and complained of mild gait disturbances and feet paresthesia for the last 12 months. Clinical examination revealed an ataxic tandem gait, abolished Achilles deep tendon reflexes, pes cavus, and mild hypopallesthesia in the lower limbs. The electrodiagnostic (EDX) study performed 1 year after symptoms onset revealed a heterogeneous demyelinating neuropathy with motor conduction blocks. (Table 1, Fig. 1A). Somatosensory evoked potentials (SSEP) indicated a delayed response in the proximal part of the upper limbs. On brachial plexus MRI, a bilateral diffuse T2 hyperintensity was found, as well as mild hypertrophy and gadolinium enhancement of the brachial plexus (Fig. 1B). Cerebrospinal fluid (CSF) analysis showed elevated protein level (1.29 g/L; *N* <0.4 g/L). Routine blood tests and next‐generation sequencing of a panel of 76 genes involved in hereditary neuropathies were negative. She was diagnosed with CIDP and received intravenous immunoglobulins (IVIg), without improvement. Two years after symptoms onset, the patient developed rapidly progressive dementia, myoclonus, and parkinsonism. Brain MRI showed thalamic and cortical hyperintensities on DWI and T2 sequences with decreased apparent diffusion coefficient (ADC). Electroencephalogram (EEG) displayed pseudo‐periodic discharges. Repeat CSF analysis showed a mildly elevated tau protein 558 ng/L (*N* <400), 14.3.3 was negative. CJD was suspected and *PRNP* analysis confirmed a heterozygous c.598G>A (p.(Glu200Lys) or E200K) mutation with a heterozygous Valine (V) / Methionine (M) codon 129 polymorphism, with a Methionine (M) on the mutated allele. Familial history reassessment identified a first cousin who died of CJD but did not undergo genetic testing. The patient died within 3 months after central symptoms onset. Postmortem neuropathological examination of the superficial fibular nerve using immunohistochemistry techniques revealed myelin alteration without lymphocyte infiltrates (Fig. 1C), associated with PrP^Sc^ aggregates in Schwann cells (Fig. 1D–F). PrP was located in myelin sheath (Fig. 1D,E) and on each side of the node of Ranvier (Fig. 1F). Neuropathological examination of the brain revealed predominantly cortico‐striato‐limbic spongiform changes, with granular and perivacuolar PrPSc deposits, as well as thickened synaptic PrP^Sc^ deposits in the cerebellar molecular layer (Fig. S1). Western blot analysis identified type 2 PrP^Sc^ (Fig. S1). The patient belonged to the M2C‐E200K subgroup as described by Baiardi *et al*.^11^

**Table 1:** Clinical and paraclinical characteristics of the patients.

Patient	Age at onset (years), gender	Delay between PNS and CNS symptoms, (months)	Symptoms at onset	Ataxia	Pin/touch hypoesthesia	Motor weakness	Osteo‐tendinous Reflex	EDX pattern, (delay from onset, in months)	Detailed NCS, myography	SSEP	Brachial plexus MRI	CSF protein in g/L (delay from onset in months)	Nerve and roots biopsy (postmortem)
1	Late fifties, F	24	Balance disturbance, feet paresthesia, postural tremor	+ (LL)	+ (LL)	−	Abolished Achilles reflex	Demyelinating neuropathy, with axonal changes in LL (12 m)	Multiples conduction blocks (max 55%), abnormal temporal dispersion, F waves prolongation, normal CV (median nerve 45–50 m/s), reduced CMAP in LL, reduced SNAP amplitude in LL	Abnormal proximal conduction in UL	Bilateral diffuse hypersignal, and hypertrophy and thickening	1.29	PrP^Sc^ in Schwann cells and DRG
2	Sixties, F	12	Balance disturbance, feet paresthesia, postural tremor	+ (LL)	+ (LL)	−	Abolished Achilles reflex	Demyelinating neuropathy, with axonal changes in LL (14 m)	Multiples conduction blocks (max 70%), abnormal temporal dispersion, F waves prolongation, reduced CMAP in LL, normal CV in (47‐51 m/sec median nerve), diffuse reduced SNAP amplitude, fibrillations in distal muscles in UL and LL	NP	NP	0.69 (14 m), 1.7 (16 m)	PrP^Sc^ in DRG

−, absence; +, presence; CMAP, compound muscle action potential; CNS, central nervous system; CSF, cerebrospinal fluid; CV, conduction velocity; DRG, Dorsal root ganglia; EDX, electrodiagnostic study; F, female; LL, lower limbs; NCS, Nerve conduction study; NP, not performed; PNS, peripheral nervous system; SNAP, sensory nerve action potential; SSEP, Somatosensory evoked potentials; UL, upper limbs.

Patient 2 was a woman in her sixties who complained of gait disturbances, feet paresthesia, and action tremor for 12 months. She had no family history. Clinical examination found an ataxic gait, distal hypoesthesia, action tremor, and abolished Achilles deep tendon reflexes. Within the next 2 months, she presented with severe gait disturbances, distal motor weakness (3/5 on the Medical Research Council scale), cognitive impairment, and parkinsonism. An EDX study performed 12 months after symptoms onset showed a demyelinating neuropathy with motor conduction blocks, abnormal temporal dispersion, and prolonged or abolished F waves, suggestive of CIDP (Table 1). Blood analyses were unremarkable. Subsequent cerebrospinal fluid analyses at 14 and 16 months after symptom onset found elevated protein levels (0.69 and 1.69 g/L, respectively), along with elevated levels of Tau (1853 and 2975 ng/L, respectively; *N* <400) and pTau proteins (83 and 79 ng/L, respectively; *N* <60). The 14–3‐3 protein was detected in the second analysis. On brain MRI, diffuse cortical and subcortical atrophy without abnormal diffusion‐weighted hyperintensities was found. EEG displayed regular triphasic waves. She died 17 months after symptoms onset. *PRNP* analysis revealed a heterozygous E200K variant with a heterozygous Valine (V)/Methionine (M) codon 129 polymorphism. Postmortem neuropathological examination of the dorsal root and DRG showed punctiform para‐axonal labeling with the anti‐PrP antibody, as observed in patient 1. Brain examination using immunohistochemistry showed plaque‐like and perivacuolar PrP^Sc^ deposits predominantly in neocortical areas and cerebellum, and thickened synaptic PrP^Sc^ deposits in the cerebellar molecular layer (Fig. S2). Western blot analysis identified type 2 PrP^Sc^ (Fig. S2). The patient could not be accurately classified into the subgroups proposed by Baiardi *et al*., as we were unable to determine the codon 129 genotype on the mutated allele.^11^ In this patient, the brain neuropathological examination showed features consistent with the M2C subgroup, including perivacuolar PrP^Sc^ deposits in the neocortex and thickened synaptic PrP^Sc^ deposits in the cerebellar molecular layer, but without the genotype evidence.

## Discussion

These cases highlight several interesting features of CJD‐related neuropathy: (i) PNS symptoms can appear up to two years before CNS features; (ii) it exhibits a particular neurophysiological profile with heterogeneous demyelination and motor blocks, and can be associated with elevated CSF protein levels, delayed proximal SSEP and plexus hypertrophy, which are supportive criteria of CIDP; (iii) it is associated with the presence of PrP^Sc^ in the PNS, notably in Schwann cells.

In previously reported cases of E200K CJD‐related neuropathy, the longest delay between peripheral and central signs was eight months.^6^ The clinical history of patient 1 suggests the possibility of a longer delay between PNS and CNS manifestations. In another genetic form, due to the Y163X variant, peripheral manifestations can be even earlier. In these patients, autonomic dysfunction and progressive, length‐dependent, predominantly sensory, axonal polyneuropathy can develop in their thirties, 10 years before cognitive disorders.^11^

The dissociation between mild clinical symptoms and marked abnormalities observed in patient 1 on the EDX study is a typical feature of genetic neuropathies, as observed in Charcot–Marie‐Tooth (CMT) disease. The most striking electrophysiological feature was the heterogeneous pattern of demyelination with motor blocks. This distinctive pattern is often associated with acquired inflammatory neuropathies, but can also be found in CMT patients (especially with *LITAF*, *MPZ*, *GJB1* and *SH3TC2* genes), and has been previously reported in CJD patients with E200K mutations.^6^, ^12^ Both patients fulfilled the electrodiagnostic criteria for CIDP, and had supportive, although non‐specific, features: elevated CSF protein, and delayed proximal SSEP, mild plexus hypertrophy and gadolinium enhancement in patient 1. Misdiagnosis of CJD‐related neuropathy as CIDP or late‐onset CMT disease may thus be a pitfall in clinical practice.^6^, ^9^ In a patient with demyelinating neuropathy, the presence of cognitive disorders, myoclonus, lack of response to immunomodulatory treatments, and a family history of CJD should lead clinicians to consider a CJD‐related neuropathy. Apart from demyelinating neuropathy, other patterns of PNS involvement such as axonal sensorimotor neuropathy have been associated and more frequently found with CJD, both in sporadic and genetic forms of the disease.^1^, ^2^

A neuropathological substrate of the peripheral neuropathy was found in the form of PrP^Sc^ accumulation in the PNS. In patient 1, PrP^Sc^ deposits were observed in the myelin sheath, consistent with the clinical and electrodiagnostic findings. Although demyelinating features in nerve tissue have been observed in E200K CJD patients, PrP^Sc^ deposits had previously only been reported in sporadic cases.^2^, ^4^, ^13^, ^14^ Favereaux *et al*. described a sporadic CJD case with PrP^Sc^ deposits in the Schwann cells of both myelinated and unmyelinated fibers of the superficial peroneal nerve.^13^ Baiardi *et al*. examined 21 peripheral nerve samples from 12 sporadic CJD patients, identifying proteinase K (PK)‐resistant PrP^Sc^ fragments in two samples by western blot analysis and full positive seeding activity in RT‐QuIC across all nerve samples.^2^ Therefore, our patients are the first E200K mutation carriers with evidence of PrP^Sc^ aggregates in the PNS.

The misfolded PrP^Sc^ protein stems from the physiological PrP protein (PrP^c^), which plays a key role in myelin formation and maintenance in the PNS; mice and goat models lacking PrP^c^ develop a demyelinating neuropathy.^15^, ^16^ Thus, we could hypothesize that prion replication in Schwann cells could disturb normal PrP^c^ function, triggering a demyelinating neuropathy.^17^ The fact that demyelinating neuropathy associated with genetic E200K CJD can precede by several years the onset of central neurological symptoms and is associated with PrP^Sc^ aggregates in the PNS raises the question of a potential centripetal propagation of PrP^Sc^ from the PNS to the brain in these patients.

## Funding Information

No financial assistance was received in support of the study.

## Conflict of Interest

The authors declare they have no competing interest in relation to this article.

## Author Contributions

Conception and design of the study: CD, AP, ER, DS, SH, JB, TM. Acquisition and analysis of data: CD, AP, JT, TM, KV, JZ, ST, AF, EB, JS, CL, PJ, CV, VD, DM. Drafting a significant portion of the manuscript or figures: CD, AP, JT, DS, KV.

## Supporting information


Figure S1.

Table S1.

## Data Availability

All data included in the study is available upon request from the editor.
