# Supplementary material for: Demyelinating neuropathy as the initial presentation of familial E200K Creutzfeldt–Jakob disease in two patients
Source: Ann Clin Transl Neurol. 2025 Jan 12;12(3):653–8. doi: 10.1002/acn3.52296 (PMC11920726; doi:10.1002/acn3.52296)
Supplement: Supplementary file 1 — Figure S1. [file ACN3-12-653-s002.pdf]

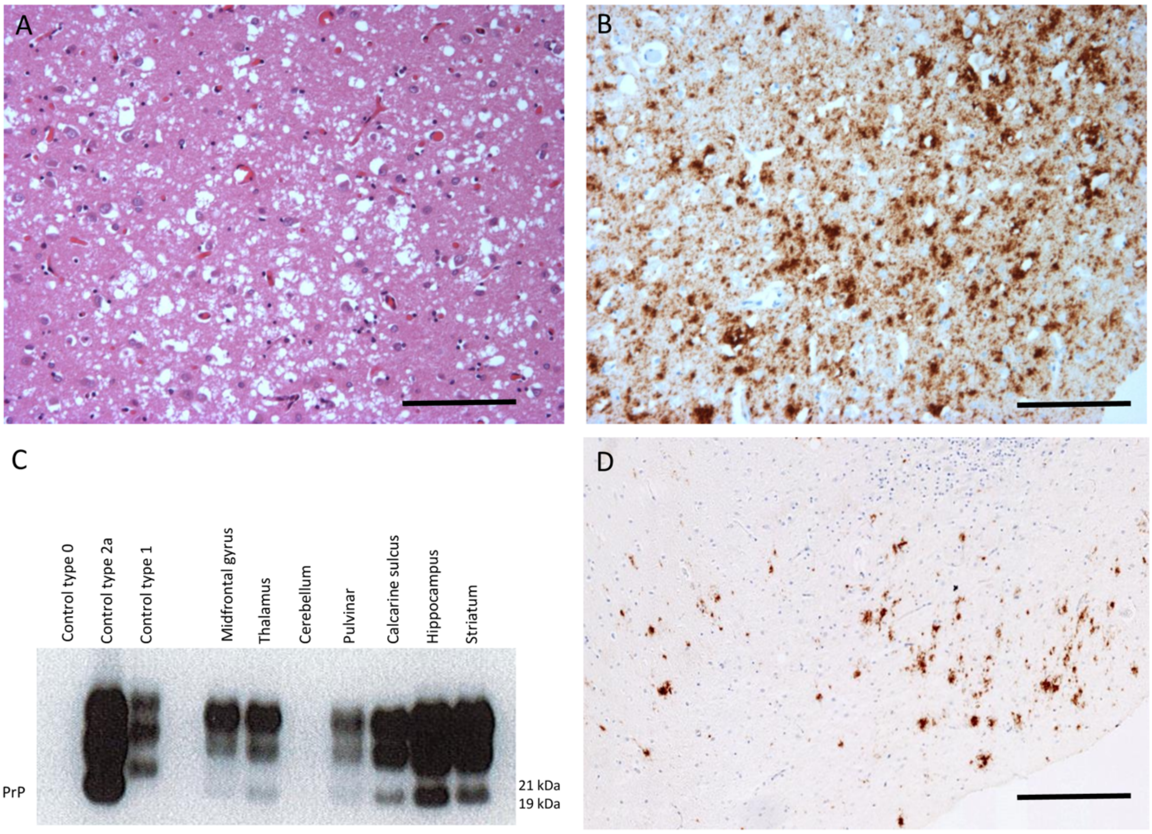

Supplementary Figure 1: Neuropathological diagnosis of Creutzfeldt-Jakob disease in patient 1.

A: H&E staining of confluent cortical spongiosis. B: PrP positive perivacuolar deposits in the cortex. C: PrP Western Blot showing a type 2 migration. D: Thick synaptic deposits in the molecular layer of the cerebellum. Scale bars = 80 µm.

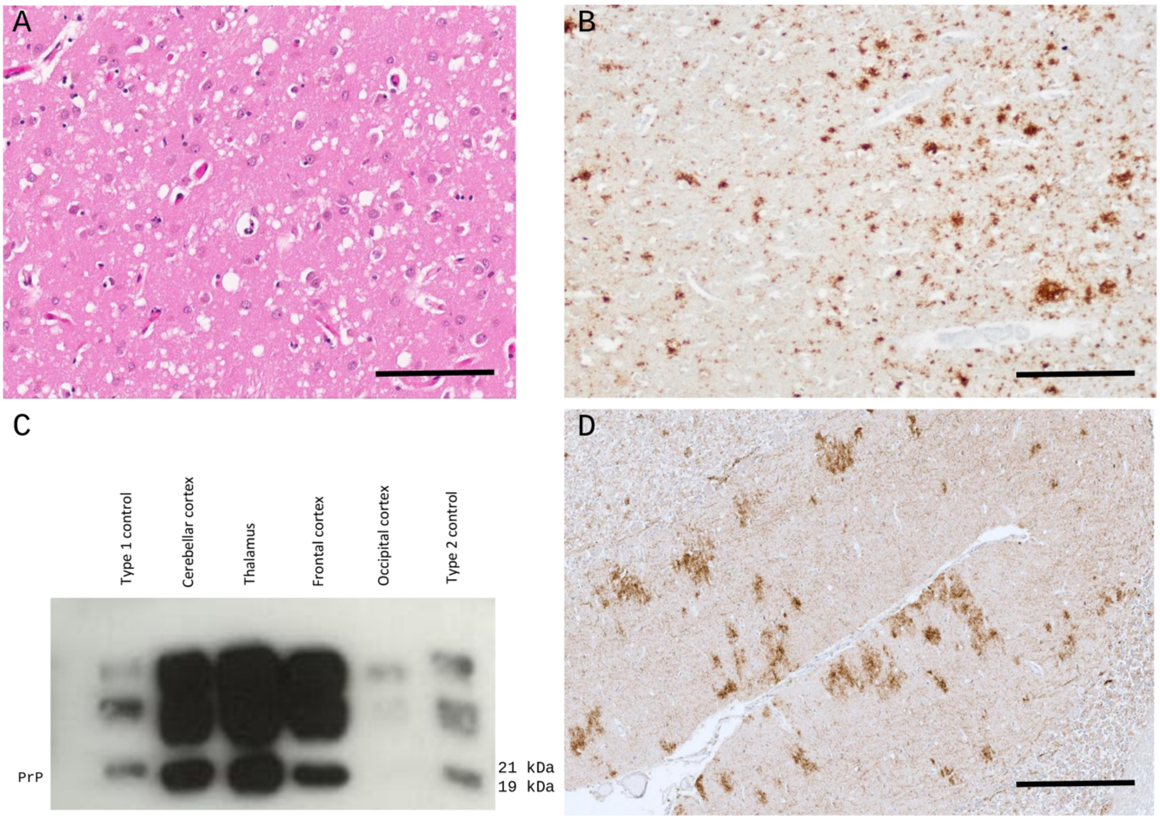

Supplementary Figure 2: Neuropathological diagnosis of Creutzfeldt-Jakob disease in patient 2.

A: H&E staining of confluent cortical spongiosis. B: PrP positive perivacuolar deposits in the cortex. C: PrP Western Blot showing a type 2 migration. D: Thick synaptic deposits in the molecular layer of the cerebellum. Scale bars = 80 µm.
