# Supplementary material for: Demyelinating neuropathy as the initial presentation of familial E200K Creutzfeldt–Jakob disease in two patients
Source: Ann Clin Transl Neurol. 2025 Jan 12;12(3):653–8. doi: 10.1002/acn3.52296 (PMC11920726; doi:10.1002/acn3.52296)
Supplement: Supplementary file 2 — Table S1. [file ACN3-12-653-s001.docx]

**Supplementary Table 1: Nerve conduction study**

|  | **Patient 1** | | | | | **Patient 2** | | | | |
| --- | --- | --- | --- | --- | --- | --- | --- | --- | --- | --- |
| Nerves | Distal latency (ms) | Ampli-tude* | Conduc-tion velocity (m/sec) | Conduc-tion block  (% amp-litude) | F min latency (ms) | Distal latency (ms) | Ampli-tude* | Conduc-tion velocity (m/sec) | Conduc-tion block  (% amp-litude) | F min latency (ms) |
| Motor nerves | | | | | | | | | | |
| Median R | *3.5* | 6.83 | *40* | **-72** | **NR** | *4.2* | 8.9 | 47 | ***-71*** | ***47*** |
| Median L | *4.4* | 6.23 | 46 | **-60** | 39.6 | *4.3* | 8.8 | 48 | ***-62*** | ***NR*** |
| Ulnar R | 2.7 | 9.72 | 52 | **-59** | **NR** | 3.1 | 7.8 | 51 | ***-41*** | *33.5* |
| Ulnar L | 3.1 | 11.12 | 52 | **-66** | 36.1 | ND | ND | ND | ND | ND |
| Fibular R | *8.0* | *0.63* | 38 | *-37* | NR | 3.8 | *1.8* | *36* | ***-39*** | ***NR*** |
| Fibular L | *7.8* | *0.52* | 35 | *-33* | NR | ND | ND | ND | ND | ND |
| Tibial R | 5.5 | *0.65* | ND | ND | 52.3 | 5.4 | *1.1* | *36* | *-35* | ***87*** |
| Tibial L | 5.2 | *0.63* | ND | ND | 42.8 | ND | ND | ND | ND | ND |
| Sensory nerves | | | | | | | | | | |
| Radial R | 1.5 | 50.2 | 49 | - | - | 2.6 | *7* | 42 | - | - |
| Radial L | 1.7 | 49.5 | 48 | - | - | 1.4 | *11* | 63 | - | - |
| Sural R | 3.1 | *4.5* | 36 | - | - | 2.6 | *3* | 42 | - | - |
| Sural L | 3.1 | *1.8* | 35 | - | - | ND | ND | ND | - | - |
| Superficial fibular R | 2.7 | *7.8* | 35 | - | - | ND | ND | ND | - | - |
| Superficial fibular L | 2.6 | *6.6* | 35 | - | - | ND | ND | ND | - | - |

In italic: abnormal result according to our laboratory normal values; in bold and italic: electrodiagnostic criteria of demyelination according EAN/PNS 2021 criteria; -: not relevant; *: amplitude in mV for motor nerves and in µV for sensory nerves; L: left; ND: Not done; NR: Not recorded; R: right
